# Multi-centre cluster randomised trial comparing a community group exercise programme with home based exercise with usual care for people aged 65 and over in primary care: protocol of the ProAct 65+ trial

**DOI:** 10.1186/1745-6215-11-6

**Published:** 2010-01-18

**Authors:** Steve Iliffe, Denise Kendrick, Richard Morris, Dawn Skelton, Heather Gage, Susie Dinan, Zoe Stevens, Mirilee Pearl, Tahir Masud

**Affiliations:** 1 Department of Primary Care & Population Health, University College London, Rowland Hill St, London NW3 2PF, UK; 2 Division of Primary Care, University of Nottingham, Tower Building, University Park, Nottingham, NG7 2RD, UK; 3 School of Health, HealthQWest, Glasgow Caledonian University, Cowcaddens Road, Glasgow, G4 0BA, UK; 4 Department of Economics, University of Surrey, Guildford, GU2 7XH, UK; 5 Department of Health Care for Older People, Nottingham University Hospitals NHS Trust, Nottingham, UK

## Abstract

**Background:**

Regular physical activity reduces the risk of mortality from all causes, with a powerful beneficial effect on risk of falls and hip fractures. However, physical activity levels are low in the older population and previous studies have demonstrated only modest, short-term improvements in activity levels with intervention.

**Design/Methods:**

Pragmatic 3 arm parallel design cluster controlled trial of class-based exercise (FAME), home-based exercise (OEP) and usual care amongst older people (aged 65 years and over) in primary care. The primary outcome is the achievement of recommended physical activity targets 12 months after cessation of intervention. Secondary outcomes include functional assessments, predictors of exercise adherence, the incidence of falls, fear of falling, quality of life and continuation of physical activity after intervention, over a two-year follow up. An economic evaluation including participant and NHS costs will be embedded in the clinical trial.

**Discussion:**

The ProAct65 trial will explore and evaluate the potential for increasing physical activity among older people recruited through general practice. The trial will be conducted in a relatively unselected population, and will address problems of selective recruitment, potentially low retention rates, variable quality of interventions and falls risk.

**Trial Registration:**

Trial Registration: ISRCTN43453770

## Background

The health benefits of physical activity have been extensively reviewed and evidence suggests that it reduces the risk of cardiovascular disease, type 2 diabetes, osteoporosis and certain cancers [1]. There is growing evidence of the association between regular physical activity and a reduced risk of all cause mortality [2], and of the potential savings for NHS budgets from exercise promotion for older adults [3]. Sedentary behaviour increases the risk of dependence, falls and fractures. Sustained levels of physical activity in adulthood maintain bone strength and can prevent fragility fractures in later life. Research has shown that a lifetime's history of regular physical activity can reduce the risk of hip fracture by up to 50% and much of this benefit is thought to result from a reduction in falls [4]. It is now clear that habitual physical activity and improved access to exercise opportunities is an important public health approach to the prevention of functional decline that can lead to frailty, falls and fractures [5].

Falls are common in people aged 65 years and older and can have serious consequences, including injury, pain, impaired function, loss of confidence in carrying out everyday activities, loss of independence and autonomy, and even death [6,7]. There is evidence that interventions providing some form of exercise may be effective in preventing falls amongst older people [8] and that healthcare costs [9,10] can be reduced if falls are reduced [11-15].

Current recommendations for health benefits are that people do at least 30 minutes of physical activity of moderate intensity on at least five days of the week [16]. However, surveys have consistently shown a high prevalence of physical inactivity in the UK population [17]. A recent systematic review comparing seventeen randomised controlled trials with different interventions designed to encourage sedentary, community dwelling adults to do more physical activity [18] concluded that interventions were effective in the short and mid term, at least in middle age, and that there were no significant increases in adverse events in the four studies that reported them. However, it is unclear which individual interventions (e.g. home-based or facility-based) are the most effective in increasing physical activity in the long term, or in specific groups (e.g. older people).

The NHS is attempting to address the problem of inactivity in a variety of ways, including exercise referral schemes in primary care ('exercise on prescription') which are currently provided by approximately 89% of Primary Care Trusts and usually involves referring patients to local leisure centres [19]. Although exercise on prescription has been shown to be feasible and effective in vulnerable older people [20], there appear to be significant barriers to the uptake of exercise classes in leisure centres. For many older people home exercise or group exercise in non-intimidating environments (e.g. community halls) may be more appealing, and result in higher uptake of exercise programmes and longer continuation of exercise. Peer activity mentors have also been shown to be effective in increasing uptake and adherence to exercise [21-24].

There are currently two existing exercise programmes, designed for use in community settings, specifically for people aged 65 and over. The first is a home based programme known as the Otago Exercise Programme (OEP) and the second is a community based group exercise programme known as the Falls Management Exercise Programme (FaME).

The OEP (Otago Exercise Programme) [25-31] and FaME (Falls Management Exercise) programmes [32] were both designed for use in community settings. specifically for people aged 65 and over. As well as being designed to reduce falls, both are based on the components of fitness and principles of programming for all older adults (i.e. warm up, mobility, stretches, strength and balance, endurance and a cool-down) and have all the elements of training appropriate for that age group. Exercises are tailored to the individual's ability and health need. Both programmes involve strength & balance training which is tailored to the individual's ability and health need.

The OEP is a home based exercise programme for older people which is effective in reducing the falls and fall-related injuries, improving balance, strength and confidence in performing everyday activities without falling, and has been shown to be cost effective for people aged 80 and over [25-31]. It was designed to be delivered by physiotherapists and nurses trained and supervised by physiotherapists. A one year evaluation of the OEP showed considerable improvements in outdoor activities (walking, shopping, gardening, other outside leisure activities) after 6 months (unpublished data Campbell) with participants continuing to exercise after completing the programme. It also showed significant improvements in executive function after 6 months [30]. Whilst the OEP has been evaluated in four controlled trials of older primary care patients in New Zealand and one RCT in Canada, it has not been tested in a primary care setting in the UK for its feasibility, impact, acceptability and cost-effectiveness.

FaME is a group exercise programme which was developed and tested in a controlled trial in the UK [32], but not in a primary care population. It aims to improve balance [33] and was designed to be delivered by qualified postural stability instructors (PSIs) [34]. It has been shown to be effective in reducing falls and injuries resulting from falls [32]. Good compliance was demonstrated with the FaME programme and nearly two thirds of people participating in FaME continued in group exercise programmes for over a year after trial completion. (unpublished data Skelton). FaME now needs to be evaluated for its impact, acceptability and cost-effectiveness within primary care.

This trial aims to fill the gaps in the current evidence base by evaluating the delivery, impact, acceptability and cost-effectiveness of a community based exercise programme (FaME) and a home based exercise programme (OEP) supported by similarly aged (peer) mentors, compared with usual care for primary care patients. The underlying assumption is that the exercises will produce sufficient subjective well-being and improved mobility to encourage continuation of higher levels of physical activity after the cessation of the intervention. Each exercise programme will be compared with usual care for effectiveness in producing sustained change in physical activity. The two programmes will be compared for cost-effectiveness if both are effective in promoting sustained change in physical activity. The primary hypotheses are: 1) Both exercise programmes produce sustained changes in physical activity (PA) compared with usual care, and 2) The Otago programme (OEP) is more cost effective than FaME

## Objectives

The primary objective is to determine the effect of two evidence based exercise programmes designed for older people, compared with usual care (i.e. with no special interventions to promote physical activity) on the achievement of recommended physical activity targets 12 months after cessation of intervention.

The secondary objectives are to:

(1) determine the health benefits of the programmes to participants starting at various levels of physical activity, particularly the effects on physical and psychological status, health status, health-related quality of life and quality adjusted life years (QALYs).

(2) estimate the costs of the exercise interventions, and possible cost offsets, and to assess the cost-effectiveness of community group exercise, and home-supported exercise compared with each other, and with usual care.

(3) determine the acceptability of the programmes, adherence rates, enabling factors and barriers to future implementation.

(4) compare the time course of responses by participants in terms of exercising at the recommended levels, at 0, 6, 12, 18 and 24 months after cessation of the intervention, between those undergoing the exercise programmes, and those receiving usual care.

(5) determine participants' perceptions of the value of exercise, and the predictors of continued exercise.

## Design/Methods

**Figure 1:**
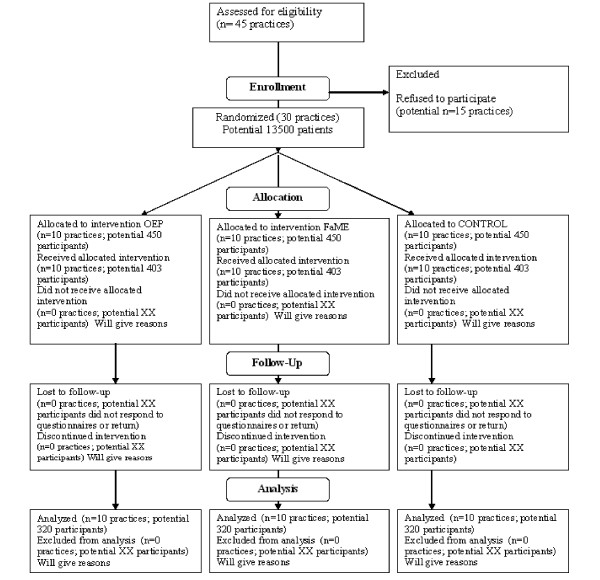
**Consort flowchart for cluster randomised trials; potential flow of participants**.

A 3 arm parallel design cluster controlled trial using minimisation for allocation at the level of general practice in two centres (London and Nottingham/Derby), comparing community-centre based group exercise programme (FaME), with a home based exercise programme and walking plan (OEP) and with usual care. There will be two years' follow-up to determine the impact, acceptability and adherence to the programme, longer term continuation of exercise and cost-effectiveness. The CONSORT diagram [35] summarises the design (Fig 1).

### Participants

Participants will be patients aged 65 years and over registered with participating general practices who give informed consent to participate.

#### Inclusion criteria for practices

Inclusion criteria will be a commitment to participate over the duration of the study and the availability of a suitable community venue in the practice catchment area.

#### Inclusion criteria for participants

Those aged 65 years and older, who can walk around independently of personal help both indoors and outdoors (with or without a walking aid) and who would be physically able to take part in a group exercise class, will be eligible to participate.

#### Exclusion criteria for participants

Those with any of the following criteria will be excluded:

• Three or more self-reported falls in the previous year;

• Resting BP > 180/100 mmHg; tachycardia > 100 bpm; those considered by their GP to have uncontrolled hypertension; significant drop in BP during exercise recorded in the medical records or found at initial assessment;

• Psychiatric conditions which would prevent participation in an exercise class, e.g. psychotic illness;

• Uncontrolled medical problems, which the GP considers would exclude patients from undertaking the exercise programme; e.g. acute systemic illness' such as pneumonia, poorly controlled angina, acute rheumatoid arthritis, unstable or acute heart failure;

• Conditions requiring a specialist exercise programme, e.g. uncontrolled epilepsy, significant neurological disease or impairment; unable to maintain seated upright position or unable to move about independently indoors;

• Not living independently (e.g. residential or nursing care);

• Significant cognitive impairment (unable to follow simple instructions);

• Already receiving long term physiotherapy or already in an exercise programme.

Exclusion criteria will be checked at the recruitment appointment by the researcher. This will include measurement of resting BP and pulse and completion of a health questionnaire. The participant's GP will be asked to confirm eligibility for each potentially eligible participant.

#### Recruitment of practices

General practices will be recruited from the Primary Care Research Networks (PCRN) in London and Nottingham/Derby. The PCRNs will be asked to identify potential participant practices by size and deprivation status. Approaches by mailed invitation, telephone contact with practice managers, and personal contact with local GP opinion leaders all be used as necessary [36].

#### Recruitment of participants

Practices will produce a single numbered list of patients aged 65 and over. Practice staff will be allowed to make and justify their own exclusions in liaison with the research team. The research team will provide the practices with a random number list to select the sample of patients to be approached after exclusions have been made. The sampling will vary depending on practice size. In practices with fewer than 600 patients aged 65 and over, all patients aged 65 and over will be invited to participate. In larger practices random sampling will be used to identify 600 patients aged 65 and over who will be invited to participate. Patients will then be sent invitation letters about the trial by their usual General Practitioner.

### Interventions

There are 3 arms to the trial:

(1) home based exercise programme and walking plan (OEP)

(2) community-centre based group exercise programme (FaME)

(3) usual care

#### Home based exercise programme (OEP)

This consists of a 30 minute programme of leg muscle strengthening and balance retraining exercises progressing in difficulty to be performed at home at least three times per week, and a walking plan to be undertaken at least two times per week for 24 weeks. Each participant will receive an instruction booklet and ankle cuff weights (starting at 0.5 kg) to provide resistance for the strengthening exercises. The programme will be introduced to participants by trained research staff, at an appropriate starting level determined at an initial assessment. Trained peer mentors will contact and visit the participants at home to start the exercise programme with them and will follow-up with up to three more home visits (as the participants require). Participants will record the days they complete the programme, and mentors will telephone them fortnightly as mentor support has been shown to be effective in increasing adherence [21-23]. Mentors will record and report any problems encountered with the exercise programme to the research team using an adverse event form developed for the study.

The delivery of the OEP will be standardised through training of research staff before the trial starts and there will be regular contact with the participants and peer mentors to check delivery protocols are being followed.

#### Community based exercise programme (FaME)

The FaME programme comprises one hour-long PSI-delivered group exercise class in a local community centre for a maximum of 15 participants, and two 30 minute home exercise sessions (based on the OEP) per week for 24 weeks. Participants will be advised to walk at least twice per week for up to 30 minutes at a moderate pace. The programme includes leg muscle strengthening and balance retraining that progress in difficulty. Progressive trunk and arm muscle strengthening, bone loading, endurance (including walking) and flexibility training, functional floor skills (see below) and adapted Tai Chi complete the evidence based programme. Ankle cuff weights, Therabands (elastic resistance training bands) and mats are also used throughout the programme. The group exercises include retraining of the ability to get up from the floor and floor exercises to improve strength, balance and coping strategies to reduce the risk of complications resulting from a long lie [34].

The delivery of the FaME programme will be standardised through training of PSIs before the trial starts and there will be regular quality assurance visits for the FaME classes to check delivery protocols are being followed.

The PSI will keep a register of attendance and record tailoring of the programme and any feedback from participants. They will follow up non-attenders by telephone as necessary, recording any positive or negative feedback, and notify the research team about reasons for non-attendance or drop-out. Participants will be given a booklet containing their home exercise instructions.

FaME groups will contain 9 or 10 participants, so there will be 4 or 5 classes per week for each of the practices allocated to this arm. The number of PSIs running these classes will be determined by their availability, but the aim is to maximise continuity and standardisation in physical activity training, so the ideal would be to have one PSI who would lead all groups in one practice. This may be difficult to achieve, but we will report the actual deployment of PSIs in our findings. The same applies to peer mentors for OEP classes

General practitioners in participating practices allocated to either the FAME or OEP programmes will be discouraged from referring participants involved in the trial to other exercise therapy projects, outside the study.

#### Usual care

Participants in the usual care arm will not be offered either the OEP or FAME programmes. They will be free to participate in any other exercise as they would if they were not participating in the trial.

#### Cultural and ethnic sensitivity

Cultural and religious requirements will be accommodated within the exercise programmes. The recommendations from the Help the Aged Minority Ethnic Elders Falls Prevention Programme (MEEFP:http://www.helptheaged.org.uk/meefp) will be followed and the research team will liaise closely with Skills Active (Sector Skills Council for Active Leisure and Learning), who are working with the Integrated Fitness Initiative's (IFI) current programme: Physical Activity Provision for Ethnic Minority Groups. In particular the FaME group classes will ensure that recommendations for attire will respect cultural, religious beliefs and customs for a range of ethnic groups. Single sex exercise groups will be scheduled as required, and separate changing facilities and gender specific instructors will be provided wherever possible. Windows in the exercise classrooms will be screened as appropriate. Family support will be encouraged, and classes will be provided at different times of the day The OEP programme will respect participant's preferences regarding family support and participation in the home exercise programme.

All research material and exercise manuals will use a maximum reading age of 9 years. Inability to read the material is not a formal exclusion criterion as the individual may be able to follow movement and correction accurately in classes and family members will be allowed to act as interpreters. Where possible, translations of research material and exercise manuals will be provided.

### Outcome measures

The primary and secondary outcome measures have been chosen to reflect the needs of participants (e.g. functional outcomes, falls, confidence, quality of life, participant costs), of commissioners of exercise services in primary care and policy makers (e.g. physical activity, falls, NHS costs).

The primary outcome will be the proportion reaching the recommended physical activity (PA) target of at least 30 minutes of activity of moderate intensity on at least 5 days each week, measured using the CHAMPS, PASE and PHONE_FITT questionnaires. While measures will be taken at 0, 6, 12, 18 and 24 months after the intervention, our primary analysis will be of 12 month data, as this is the time when the effect of the intervention is expected to be maximal, as seen in a previous evaluation of the OEP in New Zealand [37].

The secondary outcomes will include:

(1) The direct health benefits: i.e. functional and psychological status, the rate of falls (the major safety outcome measure), the number and nature of falls, and fear of falling,

(2) Stage of change, self-efficacy for exercise and physical self-perception (self-esteem relative to the physical domain), which includes measurement of perceived importance (the degree to which participants value their physical condition, body image and physical strength) to inform predictors of exercise adherence and continuation, and participants' judgement of the value or importance of physical activity,

(3) Health - related quality of life and Quality adjusted life years (QALYs) [38],

(4) The NHS and private (participant) costs of each exercise programme, and possible cost offsets, identified from a comparison of health and social service utilisation of participants in all groups during the study period.

#### Ascertainment of outcomes

The following functional assessments will be used by researchers at baseline and at the end of the interventions (and at 6 months after allocation in the usual care arm):

1. Modified Clinical Romberg Static Balance test, eyes open and closed [39].

2. Timed get-up and go (with and without distraction) (TUG) as a measure of balance and falls risk [40].

3. Functional Reach as a measure of balance and falls risk [41].

4. 30 second chair rise as a measure of lower limb strength and power [42].

The following validated tools will be used at baseline, and as self-completion questionnaires at follow-up:

1. Confidence in balance measured by the ConfBal scale [43]. A total score is provided as a measure of confidence.

2. Confidence in carrying out a range of basic activities of daily living without falling measured by the Falls Efficacy Scale-International (FES-I) [44].

3. Readiness to change measured by the transtheoretical model [45] and applying it to exercise behaviour to determine perceived barriers [46] and self efficacy for exercise [47].

4. Quality of life will be measured using Older People's QoL Questionnaire (OPQOL) [48-50]

5. Social network size and density will be measured using the brief Lubben Social Network scale [51] and perceived social support measured by the Multidimensional Scale of Perceived Social Support (MSPSS) [52].

6. Subjective Habitual Physical Activity will be assessed using a number of validated questionnaires to ensure all domains of activity and sport are considered, including the Phone-FITT, PASE and CHAMPS [53-55] and the current level of activity questions used in the Household Survey [56].

7. Attitudes and beliefs about falls prevention interventions will be measured using the AFRIS questionnaire [57].

8. Falls risk will be measured by the Falls Risk Assessment Tool (FRAT) [58].

9. Health- related quality of life will be measured by the SF-12 [59]. Quality Adjusted Life Years (QALYs), which are the main outcome for the economic analysis, will be based on EQ-5D utility weights obtained by transforming SF-12 scores [38].

In addition, demographic information, co-morbidity, medication, use of general practice and hospital and community social services will also be recorded at baseline and updated at subsequent assessments. Falls will be ascertained by self-completed fall diaries, with follow up of non-responders and telephone contact with fallers to ascertain the type of fall and any injury and health care usage that resulted.

Feedback will be sought from all exercise participants using a questionnaire which will include open questions eliciting views of the programme, reasons for drop-out, barriers to attendance and self perceptions of the benefits and disadvantages of the programmes to aid future implementation.

For the purposes of the economic analysis, the resources used in the delivery of the interventions will be collected from records kept by PSI instructors (FaME) and the research staff and peer mentors (OEP). The use of facilities and equipment, and the time spent on travel and instruction will be included and monetary costs will be assigned according to market rates.

In addition, the use of health and social care services (GP, community, outpatient, hospital admission) will be recorded for participants in all groups by means of the falls diaries. Self reported service utilisation will be verified from the primary care medical records of consenting patients after the follow up period. Costs of services will be obtained from local and national sources [60]. Health and social care costs in the exercise groups will be compared with each other and with the usual care (no exercise) group to assess the extent to which the costs of the exercise intervention may be offset by savings elsewhere in the health and social care system.

No other encouragement to continue with physical activity will be given to participants, and all potential reinforcements in the form of diaries and six monthly contracts will be given to all three arms of the trial.

#### Baseline data collection

Baseline assessment will include all functional assessments plus administration of all questionnaires described above.

#### Follow-up data collection

Follow up assessments occur at 24 weeks after the commencement of the intervention, and at 6, 12, 18 and 24 months after the completion of the intervention for participants in both intervention arms, and at 24 weeks after randomisation and at 6, 12, 18 and 24 months after completion of the 24 week assessment in the control arm.

The 24 week functional assessment will be identical to the baseline assessment plus administration of all questionnaires described above.

Assessments at 6, 12, 18 and 24 months after completion of the intervention or after completion of the 24 week assessment in the control arm will comprise postal administration of the questionnaires described above.

The primary end-point will be the proportion reaching the recommended physical activity (PA) target of at least 30 minutes of activity of moderate intensity on at least 5 days each week, measured using the CHAMPS, PASE and PHONE_FITT questionnaires, at 12 months after intervention.

### Sample size

Sample size estimates are based on the numbers of participants needed to detect differences in proportions of participants in intervention and control groups:

1) Participating in physical activity (defined as reaching the national target recommendations of five sessions of 30 minutes or more of at least moderate activity per week).

2) Self perceived health as measured by the EQ-5D index, from which meanQALY scores and the incremental cost-effectiveness ratio will be calculated.

Under individual randomisation, sample size calculations for a small effect size (0.3) [61] equivalent to a mean difference of 0.05 in the EQ-5D index in general community samples requires 176 participants per study group [62]. Published evidence of participants in a cluster randomised trial of physical activity promotion showed the proportions of participants achieving the same recommended targets for physical activity to be 14.6% (intervention subjects) vs. 4.9% (control subjects) [63]. A total of 215 participants in each study group are required to detect this difference between study groups with 90% power (5% 2-sided significance). Current plans seek a 1% increase in the number of people achieving the physical activity target of five sessions of 30 minutes or more of at least moderate activity per week, year on year [1].

Data from 24 general practices in the British Regional Heart study suggested that an intra-class correlation coefficient (ICC) not exceeding 0.02 was appropriate for physical activity outcomes among middle aged men, but this study aimed to represent the full range of cardiovascular disease prevalence across Britain and the range would probably be less in the proposed study as it is less geographically dispersed [64]. Also ICCs collected for a range of variables in primary care settings have typically averaged 0.01 [65].

Based on an intra class correlation coefficient of 0.01 the design effect would be 1.31, because 32 participants will provide data per practice (see next paragraph). If 215 participants per arm are required (before allowing for attrition) for an individually randomised design (90% power, 5% 2-sided significance), 282 per arm would be required for the clustered design. Allowing for 30% attrition, this equates to 403 per arm. The sample size is based on detecting differences between each intervention (exercise programme) and the control arm, and there is unlikely to be enough power to detect modest differences in outcome between the two intervention arms.

Assuming an average practice size of 6000 patients, 15% (900) of whom are aged 65 and over [66] and a random 1 in 2 sample (ratio will vary according to the practice size) of patients are approached to take part in the study, 600 patients aged 65 and over would be approached. Assuming that approximately 45 of these patients per practice agree to participate, and allowing for an attrition rate of 30%, outcome data would be obtained on 32 participants per practice.

It is expected that all or most patients in each practice will be invited to join the trial. In larger than average practices, however where the patient list is very large, a stratified random sample of 600 patients will be drawn. Response rates from each practice will be recorded.

### Randomisation

Due to the relatively small number of practices in the trial, minimisation will be used to allocate practices to treatment arms to ensure maximum balance [67]. After all participants from a practice have been recruited, their practices will be individually allocated to a study arm by the London co-ordinating centre. Practices will be given an identification number and treatments will be assigned, by the senior statistician for the trial, using computer generated random number tables, embedded in a computer programme for minimisation. The variables to be used in the minimisation process will be trial centre (London/Nottingham & Derby), practice size (>= median practice size/< median practice size) and the index of multiple deprivation 2007 [68] (>= median IMD2007/< median IMD2007). Minimisation will be undertaken using the MINIM program [69]. Practice recruitment and allocation will be performed concurrently in the two centres. Median practice size and IMD2007 values for the whole of England will be used as cutpoints for the minimisation process.

### Concealment of allocation

Practices are allocated to intervention or usual care only after all patients are recruited. The practices, their patients and the researchers undertaking baseline assessments are all blinded to allocation until this point.

### Blinding

It is difficult for participants to be blind in trials of exercise interventions, and for researchers to be blind to the allocation of participants as they will recruit participants and undertake baseline and follow up assessments. The researchers assessing outcomes are not blinded for pragmatic reasons only; the study is funded to support only enough researchers to carry our recruitment and follow-up simultaneously. However, general practices and their participants, and researchers having contact with practices and participants will not have foreknowledge of the treatment arm allocation of the practice, which will not be disclosed until after all participants within a practice have been recruited. Also, for the statistical analysis of participants' data, the statistician will be blind to the treatment arm allocation of the practices.

### Withdrawals

Participants may be withdrawn from the trial either at their own request or at the discretion of the Investigator after discussion with the chair of the trial steering committee. Participants will be made aware (via the information sheet and consent form) that withdrawal from the trial will not affect their future care, and that the data collected to date may still be used in the final analysis. Any requests to withdraw data made by individuals withdrawing from the trial will be respected. The research teams at each site will advise discontinuation of exercise intervention or withdrawal from the trial if the exercise intervention poses a hazard to the safety of a participant, or if the participant poses a hazard to the safety of another participant. Those who withdraw from the trial will not be replaced.

#### Contamination

Usual care arm participants may be disappointed and seek their own way of increasing physical activity, but the falls and service use diaries and the 6 monthly reviews will capture this information.

### Statistical methods

Characteristics of participants and practices will be compared descriptively at baseline. Comparisons between treatment arms will be made using random effects models to allow for clustering between practices. Linear regression models will be used for continuous outcome variables, logistic models for binary outcome variables (in particular the primary endpoint, namely attainment of recommended exercise level at 12 months after the intervention), and Poisson or negative binomial models for data on rate of falls. The assumptions for using each model will be checked and analyses adjusted accordingly. All analyses will be undertaken, adjusted (a) for variables used for minimisation (centre, deprivation and practice size), (b) for baseline values of the outcome measure, and (c) for baseline variables which differ to a clinically significant extent between groups. Differential effects of the intervention by age, sex and baseline activity levels will be assessed for the primary outcome measures by adding terms for the interaction between age, sex and baseline activity levels and treatment arm to the regression models.

Multilevel models will be applied to take account of clustering at the practice level (applicable to all arms of the study) and practitioner effects which will apply to differing extents in the OEP arm (due to the effectiveness of different PSIs) and FaME arm (due to the effectiveness of different PMs). Analyses as recommended by Roberts [70] and Walwyn and Roberts [71] (2009) will be applied for this purpose.

While the primary endpoint will be attainment of recommended exercise levels at 12 months, we will investigate the profile of exercise attainment at all time points (0, 6, 12, 18 and 24 months post intervention). We will carry out repeated measures analysis using generalised estimating equations which account for the intra-participant dependency of the repeated measures. In particular we will investigate evidence for differences in effect of the interventions at the different time points.

As the study consists of two intervention arms and one control arm, primary analysis will consist of comparing each intervention group with the control group. No formal adjustment of p-values will be made, since the sample size has been specifically designed to test each intervention separately.

The economic analysis will adopt standard techniques of economic appraisal [72]. The measure of effectiveness will be mean differences in QALY scores at the end of follow up after adjustment for baseline values, estimated in an analysis of covariance. If statistically significant differences between groups are found, incremental cost effectiveness ratios will be calculated to show the extra cost incurred per QALY gained. Comparisons will be conducted between the usual care group and each type of exercise programme, and between the two interventions, using group means of follow-up QALY values after adjusting for baseline levels. The impact of uncertainties in the estimation of costs and outcome variables will be explored using one way and probabilistic sensitivity analysis. Bootstrap methods will be used to represent uncertainty of estimates, either for constructing confidence intervals or probability curves.

Sensitivity analyses will investigate the cost-effectiveness of the interventions for people with different levels of physical activity, health status and health related quality of life at baseline. Secondary cost-effectiveness analyses will be conducted using physical activity and other outcomes as the measures of effectiveness.

Missing outcome data will be assumed to be "missing at random" (MAR), conditional on key predictors of "missingness" (in particular baseline values of the response variable, treatment arm, and measures of compliance post randomisation) Multiple imputation of outcome variables will be carried out using these predictors of "missingness" [73]. A further sensitivity analysis will be carried out where all who do not report their exercise levels will be assumed to be non-exercisers.

The full analysis set will comprise all randomised participants for whom one post-baseline assessment of the primary outcome measure is available. The per-protocol set will comprise all randomised participants who are deemed to have no protocol violations. CACE analysis will be carried out [74].); this seeks to estimate the difference between compliers in the intervention groups with those who would have complied in the non-intervention group. The safety set will be all randomised participants who undertake at least one OEP session or FaME class.

### Protocol violations

Participants who are randomised to the OEP or FaME programmes who do not undertake any of the OEP programme or attend any FaME classes will be deemed to be protocol violations.

### Risks

Participants will complete a health questionnaire at recruitment which is sent to their GP to confirm exclusion criteria, prior to commencement of either exercise programme. Previous evaluation of the OEP showed significant reductions in falls and injuries [31] No adverse effects occurred in previous evaluations of either the OEP or FAME programmes [32]. Safe exercise guidelines will be followed, pre-exercise assessment will be conducted and exercise intensity and difficulty will be increased with caution to minimise the risk of injury. All participants and their general practitioners will be informed of the potential risk of injury from any exercise programme in the information documents provided for participants and practices, so that consent is obtained with full knowledge of such risks.

### Adverse events

An adverse event (AE) is any unfavourable and unintended sign, symptom, syndrome or illness that develops or worsens during the period of observation in the trial. This includes:

(1) Exacerbation of a pre-existing illness.

(2) Increase in frequency or intensity of a pre-existing episodic event or condition.

(3) Condition detected or diagnosed after the intervention even though it may have been present prior to the start of the study.

(4) Continuous persistent disease or symptoms present at baseline that worsen following the start of the study.

A Serious Adverse Event (SAE) is any adverse event occurring following study mandated procedures, having received the OEP or FaME programmes or usual treatment that results in any of the following outcomes:

(1) Death

(2) A life-threatening adverse event

(3) Inpatient hospitalisation or prolongation of existing hospitalisation

(4) A disability/incapacity

(5) A congenital anomaly in the offspring of a participant

Important medical events that may not result in death, be life-threatening, or require hospitalisation may be considered a serious adverse event when, based upon appropriate medical judgment, they may jeopardize the participant and may require medical or surgical intervention to prevent one of the outcomes listed in this definition.

All adverse events will be assessed for seriousness, expectedness and causality. All adverse events will be recorded and closely monitored until resolution, stabilisation, or until it has been shown that the study intervention is not the cause.

Participants will be asked to contact the trial site immediately in the event of any serious adverse event. The Chief Investigator shall be informed immediately and shall determine seriousness and causality in conjunction with any treating medical practitioners. A serious adverse event that is deemed directly related to or suspected to be related to the trial intervention will be reported to the ethics committee.

### Informed consent

Written informed consent will be obtained from all participants. The decision regarding participation in the study is entirely voluntary. The researcher will emphasize to potential participants that consent regarding study participation may be withdrawn at any time without penalty or affecting the quality or quantity of their future medical care, or loss of benefits to which the participant is otherwise entitled. No trial-specific interventions will be undertaken before informed consent has been obtained.

### Ethical and organisational review

Ethical approval has been granted to the trial from Nottingham Research Ethics Committee 2 (application number 08/H0408/72). National Health Service (NHS) Research & Development (R&D) approval has been granted by NHS Nottinghamshire County and Westminster, Brent, Hounslow and Barnet PCTs, and will be sought from other relevant PCTs as practices are recruited to the study.

## Discussion

The ProAct65+ trial is a primary care based exercise intervention for older people with wide inclusion criteria. The pragmatic trial design replicates the approach taken in successful primary care trials in New Zealand [37,63] and differs from the majority of trials which focus on falls reduction in selected groups by having continuation of physical activity as its primary outcome.

The problems that we anticipate are: 1) biases in recruitment, with those already exercising at a relatively high level being more likely to volunteer for this trial; 2) limited retention of recruits to the study, which we hope to minimise by relatively frequent but brief contact with participants after the end of the exercise programmes; 3) variation in 'doses' of exercise promotion, which we hope to avoid through our quality assurance processes; and 4) an increase in falls risk, as in previous studies [37], which we will counter through training of staff, risk reduction and risk management programmes.

Because the trial will document the levels of activity of participants, which can be compared with population norms, and the number screened, the number who are ineligible and the number who refuse, its findings will be generalisable. The findings will therefore be important for informing policy on exercise promotion and falls prevention amongst older people. They will be relevant to older people and to policy makers working in health, social care and leisure arenas, health and social care commissioners and providers, leisure providers and charities and voluntary organisations working with older people.

## Competing interests

DS and SD are Directors for Later Life Training, who deliver FaME and OEP training to health and leisure professionals across the UK. The other authors declare that they have no competing interests.

## Authors' contributions

SI, DK, TM, SD, DS, RM and HG developed the research proposal for funding, and together with ZS, MP wrote and refined the trial protocol.
